# Assessing Discharge Readiness and Influencing Factors Among Patients with Aortic Dissection: A Cross-Sectional Study

**DOI:** 10.1097/AJN.0000000000000232

**Published:** 2026-01-22

**Authors:** Long Ling, Yanqiu Hu

**Affiliations:** **Long Ling** and **Yanqiu Hu** are clinical nurses in the Department of Cardiovascular Surgery at the First Affiliated Hospital of Soochow University in Suzhou, Jiangsu, China. Contact author: Yanqiu Hu, thzlxn@sina.com. The authors have disclosed no potential conflicts of interest, financial or otherwise.

**Keywords:** aortic dissection, discharge plan, discharge readiness, nursing, patient education

## Abstract

**Background:**

Discharge readiness is a critical determinant of patient outcomes, particularly for patients with aortic dissection, a highly lethal cardiovascular disease.

**Purpose:**

This study aimed to assess the level of discharge readiness in patients with acute aortic dissection, and to analyze the influencing factors in order to better support patients' postdischarge recovery and improve their long-term prognoses.

**Methods:**

Patients who underwent surgery for aortic dissection at our hospital and were in the process of recovery and preparing for discharge were included in the study. Discharge readiness was assessed with a widely used, validated discharge readiness scale. Multiple linear regression analysis was performed to identify influencing factors.

**Results:**

A total of 218 patients with aortic dissection were enrolled. The mean (SD) total discharge readiness score for these patients was 160.75 (21.38), and the mean (SD) score per item was 7.38 (1.02). Correlation analysis revealed that age, education level, place of residence, family monthly income, and recurrent aortic dissection were each significantly correlated with discharge readiness. Multiple linear regression analysis identified age, education level, place of residence, family monthly income, and recurrent aortic dissection as significant independent predictors of discharge readiness in patients with aortic dissection.

**Conclusions:**

This study revealed that patients with aortic dissection generally had discharge readiness scores at the lower end of the moderate range, indicating the need for improvement. It's imperative that health care providers emphasize patient education prior to discharge and develop and implement personalized discharge plans for these patients in order to improve not only their discharge readiness but also their postdischarge quality of life and long-term prognoses.

## Graphical abstract

**Figure:**
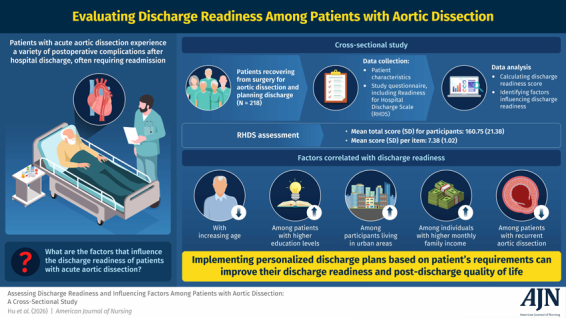

Acute aortic dissection, a serious cardiovascular condition involving a tear to the inner layer of the aorta, is characterized by rapid onset, poor prognosis, and a high mortality rate.^1^-^5^ It is concerning that, in recent years, there is evidence of a decrease in the average age of onset.^6^ Although surgical treatment techniques have advanced,^7^ the procedures remain complex and difficult, and patients are prone to a variety of postoperative complications that can significantly impact their physical and mental health.^8^ Such complications include recurrent dissection aneurysms, dissection rupture, and cardio- and cerebrovascular events including spinal cord injury and stroke, leading to high readmission rates.^2^,^9^ Research indicates that up to one-third of patients with aortic dissection are readmitted within 90 days of treatment, regardless of whether this involved endovascular or open surgical repair.^10^,^11^

Moreover, patients with aortic dissection often experience persistent postoperative symptoms such as bodily pain and impaired physical functioning, resulting in a markedly lower quality of life after discharge compared to the general population.^8^,^12^,^13^ Indeed, in one study among patients undergoing emergency surgery for aortic dissection, Zhang and colleagues found that 31.5% developed posttraumatic stress disorder, while 31.2% experienced anxiety and 26.3% experienced depression.^5^ Another study by Chaddha and colleagues among survivors of acute aortic dissection found that the rate of medication nonadherence was 36%.^14^

Discharge readiness refers to a patient's ability to safely and effectively transition from hospital care to home and society; it encompasses their physical, psychological, functional, and social health status at time of discharge.^15^,^16^ A comprehensive assessment of such readiness necessarily involves evaluating the patient's ability to perform self-care, manage their recovery and rehabilitation, and reintegrate into society.^16^ Discharge readiness is a significant factor influencing patients' postdischarge outcomes and quality of life.^17^-^19^ For patients with aortic dissection, both continuity of care and the comprehensive assessment of their discharge readiness are paramount.

Medical capabilities and treatments have continued to evolve; for example, one study among patients undergoing surgery to repair aortic dissection found that early myocardial reperfusion was associated with shorter ICU and hospital lengths of stay.^20^ Furthermore, health care systems have sought to decrease inpatient lengths of stay and increase bed turnover rates in order to reduce costs. Consequently, patients with aortic dissection are experiencing shorter hospitalizations and are discharged at earlier stages of recovery than was typical in the past. Given these circumstances, the ability of patients to manage self-care at home after discharge becomes especially important. Currently, discharge readiness and the factors that influence such readiness among patients with aortic dissection are not well understood.

**Study purpose**. This study sought to assess the discharge readiness of patients with acute aortic dissection, and to analyze the influencing factors in order to enhance clinician awareness of relevant concerns and to better support patients' postdischarge recovery and improve their long-term prognoses.

## METHODS

**Study design and setting**. This study employed a cross-sectional survey methodology and was conducted at a large teaching and research hospital in China. The research protocol was formally approved by the hospital's ethics committee. All participants provided their consent by signing a paper-based informed consent form prior to data collection.

**Sample**. The study was conducted from January 1, 2023, through November 30, 2024, focusing on patients who had undergone surgery for aortic dissection and were recovering and preparing for discharge. Patients were eligible for inclusion if they were 18 years of age or older and met the diagnostic criteria for aortic dissection as outlined by the European Society of Cardiology, had undergone surgery for this condition at the hospital, had clear consciousness, and voluntarily consented to participate in the study. Patients with cognitive or communication impairments were excluded, as were those who declined to participate.

**Data collection**. We collected the following participant characteristics: gender; age; body mass index (BMI); education level; residence; marital status; and family monthly income; as well as any history of hypertension, diabetes, hyperlipidemia, or recurrent aortic dissection. To assess participants' discharge readiness, we used the Chinese version of the Readiness for Hospital Discharge Scale (RHDS), a validated and widely used tool.^17^,^21^,^22^ The scale has demonstrated high reliability, with a Cronbach α coefficient of 0.893.^21^ It covers four dimensions: personal status (seven items), disease knowledge (eight items), postdischarge coping ability (three items), and available social support (four items), totaling 22 items. All response options used a Likert-type scale ranging from 0 to 10, with each value corresponding to a specific degree of the measured attribute. Total possible scores ranged from 0 to 220. Based on previous research, we categorized a participant's average per item score as follows: less than 7 indicates inadequate readiness, 7 to 8 indicates moderate readiness, 8 to 9 indicates high readiness, and above 9 indicates very high readiness. A higher total score indicates a higher overall level of discharge readiness.

The study questionnaire, which included the RHDS, was distributed on-site in paper form to participants on the day of discharge, based on the information recorded in the cardiovascular surgery department's discharge register. During distribution, the researchers provided the participant with a detailed description of the study's purpose and significance, and explained how to complete the questionnaire. For those who were unable to fill out the questionnaire independently due to physical limitations or other reasons, researchers provided the questions and available answer choices verbally in a neutral tone, then filled in the patient's responses on their behalf. Completed questionnaires were collected immediately. Any questionnaires that were not fully completed or contained obvious logical errors were considered invalid and excluded from analysis.

**Data analysis** was conducted using IBM SPSS version 26. General characteristics of the participants were described using counts or frequencies and percentages. Participants' discharge readiness scores were summarized using mean (SD). Before group comparisons were made, the data were tested for normality using the Shapiro–Wilk test and for homogeneity of variances using the Levene test. For two-group comparisons of normally distributed data, independent-samples *t* tests were used; for non–normally distributed data, the Mann–Whitney *U* test was applied. For multiple group comparisons, one-way analysis of variance (ANOVA) was used when data were normally distributed and had equal variances; otherwise, the Kruskal–Wallis *H* test was used. Relationships between discharge readiness and specific participant characteristics were explored using the Pearson *r* correlation coefficient for normally distributed data or Spearman rank correlation coefficient (ρ) for non–normally distributed data. Variables that showed statistical significance in univariate analysis and those found to be correlated using correlation analysis were included as independent variables in a multiple linear regression analysis, using the discharge readiness score as the dependent variable, to identify factors influencing discharge readiness. Significance was set at *P* ≤ 0.05.

## RESULTS

A total of 218 patients with acute aortic dissection were included in this study. Most of the participants identified as male, comprising 81.7% of the sample. The mean age was 54.16 years (SD, 8.77 years), and the mean BMI (calculated as weight in kilograms divided by height in meters squared) was 25.51 (SD, 2.04). About two-thirds of the participants were urban residents, comprising 62.4% of the sample. For more on participant characteristics, see Table 1.

**Table 1.** Participant Characteristics (N = 218)

Characteristic	n (%)	Discharge Readiness Mean Total Score (SD)	*t/F*	*P*
Gender			8.44	0.08
Male	178 (81.7)	159.60 (17.66)		
Female	40 (18.3)	162.94 (18.91)		
Age, years			2.40	0.01
19-39	30 (13.8)	162.10 (19.37)		
40-60	152 (69.7)	162.14 (16.86)		
>60	36 (16.5)	157.69 (18.05)		
BMI			1.76	0.10
<24	89 (40.8)	162.48 (16.44)		
≥24	129 (59.2)	161.03 (17.58)		
Highest education level			1.78	0.001
Primary school	14 (6.4)	152.38 (18.60)		
Junior high school	38 (17.4)	155.12 (19.35)		
Senior high school	52 (23.9)	160.42 (18.47)		
Associate degree	69 (31.7)	161.40 (17.33)		
Bachelor's degree or above	45 (20.6)	166.26 (18.62)		
Residence			6.43	0.02
Rural area	82 (37.6)	154.50 (17.69)		
Urban area	136 (62.4)	164.75 (17.88)		
Marital status			3.28	0.09
Married	170 (78.0)	163.54 (17.59)		
Single	28 (12.8)	159.46 (19.07)		
Divorced	14 (6.4)	158.70 (16.89)		
Widowed	6 (2.8)	159.04 (18.33)		
Family monthly income, RMB			2.45	0.01
<5,000	76 (34.9)	157.20 (17.16)		
5,000-10,000	102 (46.8)	161.36 (18.24)		
>10,000	40 (18.3)	166.62 (18.48)		
Hypertension			5.21	0.22
Yes	88 (40.4)	160.27 (16.42)		
No	130 (59.6)	163.10 (14.77)		
Diabetes			6.03	0.11
Yes	71 (32.6)	160.33 (18.06)		
No	147 (67.4)	162.18 (17.25)		
Hyperlipidemia			4.30	0.10
Yes	52 (23.9)	161.04 (18.30)		
No	166 (76.1)	162.33 (17.48)		
Recurrent aortic dissection			4.23	0.001
Yes	12 (5.5)	156.04 (18.63)		
No	206 (94.5)	161.96 (17.14)		

BMI = body mass index; RMB = renminbi, official unit of currency in China.

Regarding discharge readiness as assessed by the RHDS, the mean total score (SD) for participants was 160.75 (21.38), and the mean score (SD) per item was 7.38 (1.02). See Table 2.

**Table 2.** Participant Discharge Readiness Scores (N = 218)

Dimension	Mean Score (SD)	Mean Score (SD) Per Item
Personal status	49.18 (6.01)	7.04 (0.89)
Disease knowledge	57.55 (7.36)	7.15 (0.80)
Postdischarge coping ability	22.75 (3.89)	7.44 (1.64)
Available social support	31.27 (4.12)	7.90 (1.04)
Total score	160.75 (21.38)	7.38 (1.02)

Correlation analysis results showed that age (*r* = 0.58), education level (ρ = 0.61), place of residence (ρ = 0.57), family monthly income (*r* = 0.56), and recurrent aortic dissection (ρ = 0.62) were significantly correlated with discharge readiness. See Table 3.

**Table 3.** Correlation Analysis of Discharge Readiness Scores and Participant Characteristics (N = 218)

Characteristic	Pearson *r*	*P*
Age	0.58	0.02
BMI	0.09	0.11
Family monthly income	0.56	0.02
Characteristic	Spearman ρ	*P*
Gender	0.19	0.10
Education level	0.61	0.01
Residence	0.57	0.04
Marital status	0.18	0.18
Hypertension	0.13	0.11
Diabetes	0.19	0.08
Hyperlipidemia	0.10	0.11
Recurrent aortic dissection	0.62	0.003

BMI = body mass index.

Multiple linear regression analysis identified age, education level, place of residence, family monthly income, and recurrent aortic dissection as significant independent predictors of discharge readiness. See Table 4.

**Table 4.** Multiple Linear Regression Analysis of Influencing Factors of Discharge Readiness (N = 218)

Variable	Unstandardized Coefficient	Standard Error	Standardized Coefficient	*t*	*P*
Constant	–72.12	22.75	—	–3.22	0.006
Age	1.28	0.15	0.67	5.38	0.02
Education level	–3.87	0.20	–0.71	–4.12	0.02
Residence	1.64	0.11	0.91	4.01	0.04
Family monthly income	1.92	0.25	0.93	4.81	0.02
Recurrent aortic dissection	2.84	0.23	1.15	4.66	0.01

## DISCUSSION

The findings of this study indicate that, among patients who undergo surgery for aortic dissection, the level of discharge readiness is only moderate at best. This suggests there is significant room for improvement in preparing these patients for discharge.

Compared to discharge readiness levels reported for patients with coronary heart disease following stent implantation,^23^ the readiness level for the patients treated for aortic dissection in this study is somewhat lower. This discrepancy may be closely related to the nature of acute aortic dissection, a condition characterized by rapid onset, swift progression, and a high mortality rate.^2^,^4^ Patients often perceive a marked decline in their self-care abilities, leading to an increased need for assistance.^24^ It's important for health care providers to ensure the high quality of discharge guidance and to conduct comprehensive assessments of patients' discharge readiness. This approach can effectively reduce both the incidence of postoperative complications and the risk of readmission for these patients, thereby improving their long-term prognosis.

**Factors influencing discharge readiness**. *Age*. This study found a significant negative correlation between patient age and discharge readiness, such that as age increased, discharge readiness scores tended to be lower, a finding in keeping with results from prior research.^25^ This phenomenon may be attributed to several factors. First, with advancing age, there is generally a gradual decline in organ function, memory, and cognitive abilities, resulting in greater challenges in understanding and mastering condition-related knowledge and rehabilitation skills. Moreover, older patients are more likely to have multiple chronic comorbidities and to be more physically vulnerable than younger patients, which further increases the difficulties they face during postoperative recovery.^8^ Thus, providers should pay particular attention to the discharge readiness of older adults recovering from surgery for aortic dissection. Measures such as providing personalized health education, enhancing psychological support, and optimizing discharge guidance can be implemented to help patients better adapt to life after surgery and improve their discharge readiness.^26^,^27^ For instance, to address the issue of memory decline, the use of visual aids and repeated explanations can help patients to better understand and retain information about postoperative management and self-care. Regardless of patient age, strengthening communication with patients and families in order to better understand their needs and difficulties will allow clinicians to provide necessary assistance and optimal support, thus enhancing patient discharge readiness.

*Education level*. In this study, education level was positively correlated with discharge readiness, such that patients with lower education levels tended to have lower discharge readiness scores, a finding that aligns with the results of prior research.^25^,^28^ As a key determinant of health literacy, education influences patients' ability to comprehend and retain relevant information. Patients with higher education levels generally possess greater health literacy, enabling them to actively engage in their treatment, seek out disease information through various channels, and communicate effectively with their providers. In contrast, patients with lower education levels may face challenges in understanding medical information and adhering to medical advice, which can directly impact their discharge readiness. This underscores the need for providers to pay special attention to this patient population. To enhance their discharge readiness, providers can implement a range of strategies, such as delivering health education in plain language, using visual aids, and reinforcing patient understanding of self-management through repeated explanations and practical demonstrations.^29^,^30^ Communication with patients and families should consider the education levels of both, in order to identify needs and difficulties specific to those with lower education levels and facilitate tailored assistance and support.

*Place of residence*. This study found that participants living in urban areas exhibited greater discharge readiness than their rural counterparts. This disparity may exist, at least in part, because people residing in rural areas often live considerable distances from hospitals, delaying their access to emergency care. Furthermore, the relative scarcity of medical resources in rural areas can hinder patients' ability to obtain current, evidence-based information and guidance. To enhance patients' discharge readiness, one viable strategy may be to incorporate various means of telecommunication, from telephones to internet communication and telemedicine platforms. For example, through telemedicine platforms, health care providers can offer ongoing health education and consultation services, helping patients to better understand disease management and self-care skills.^31^ Such tailored information and support not only increase the relevance and efficacy of health education, but may also boost patients' confidence in managing their conditions. And making use of the telephone and social media platforms can facilitate patients' connections to their families and communities, enabling them to receive essential social support.

*Family monthly income* was found to significantly influence discharge readiness. Prior research indicates that, compared to people with lower incomes, people with higher incomes tend to have less distress and be more optimistic when facing serious illness, perhaps in part because they have more financial resources at their disposal.^32^,^33^ It stands to reason that having a more positive outlook could improve patients' cooperation with their providers, encourage learning about their condition, and promote more active participation in their rehabilitation, thereby enhancing their discharge readiness and adaptation to postdischarge life. In contrast, patients with lower incomes may experience increased financial stress when facing illness, which could lead to or exacerbate anxiety and depression. Such emotions could diminish psychological well-being, reduce treatment adherence, and impede recovery, lowering their discharge readiness.

Moreover, economic status can impact the availability of and access to social support. Patients with higher incomes tend to have broader social networks, enabling them to access more social support and resources.^34^ For instance, they may receive financial assistance, emotional support, and practical help from friends and family, giving them a more stable environment for recovery and easing the transition to postdischarge life. Without such robust support, patients with lower incomes may feel financial stress more acutely. To improve discharge readiness, health care institutions and society at large should collaborate to provide comprehensive and personalized support for patients of different economic backgrounds. Measures might include proactively engaging health insurers to minimize delays in obtaining approvals, improving mental health education and developing interventions to foster positive attitudes, and fostering social support networks that offer patients greater access to public as well as private resources and assistance.^34^,^35^

*Recurrent aortic dissection*. In this study, participants with recurrent aortic dissection generally had lower discharge readiness scores compared to those experiencing their first episode. Because aortic dissection is a critical condition with substantial treatment costs, patients and their families often place great importance on the initial episode, actively cooperating with medical and nursing interventions and proactively learning about both aortic dissection and rehabilitation skills. But as Li has noted, experiencing this sudden medical crisis often leads to unwanted emotions such as anxiety, fear, and depression.^36^ It's possible that patients who have recurrent aortic dissections may be even more prone to such emotions, undermining their confidence in their ability to recover. These emotions could affect not only their adherence to treatment but also their capacity for social interaction, diminishing their social support. Health care providers should closely monitor the psychological coping mechanisms of patients with recurrent aortic dissection, promote their confidence regarding recovery, and analyze the primary risk factors for recurrence in order to implement effective prevention strategies.^37^,^38^

**Limitations and further research**. This study had several limitations. First, as this was a single-site study with a relatively small sample size, the results cannot be generalized. Further research using larger sample sizes and multiple sites is needed to enhance the generalizability and applicability of the findings. Second, because of time and funding constraints, we were able to conduct only a preliminary investigation into the current status of discharge readiness and the influencing factors for patients with aortic dissection. Further research that builds on our findings can help guide the development of targeted interventions aimed at improving the discharge readiness of these patients.

## CONCLUSIONS

This study revealed that patients with aortic dissection generally had discharge readiness scores at the lower end of the moderate range, indicating the need for improvement. Five factors––age, education level, place of residence, family monthly income, and recurrent aortic dissection––were identified as significantly influencing their discharge readiness. It's imperative that interventions aimed at addressing these influencing factors be developed and implemented in personalized discharge plans. Emphasis should be placed on improving the quality of predischarge education to increase patients' discharge readiness. Concurrently, future research should continue to explore and evaluate existing and new intervention strategies. Together these measures can improve patients' quality of life after surgery for aortic dissection as well as their long-term prognoses.
